# “It’s a kind of double-edged sword”: exploring the social media-related experiences of adults with visible differences using photo-elicitation interviews

**DOI:** 10.1371/journal.pone.0324938

**Published:** 2025-05-22

**Authors:** Ella Guest, Wylde Roberts-Mills, Anna Zarola, Amy Slater

**Affiliations:** Centre for Appearance Research (CAR), University of the West of England (UWE), Bristol, England, United Kingdom; University of Minnesota, UNITED STATES OF AMERICA

## Abstract

**Objectives:**

Visible differences are associated with experiences of stigma, discrimination, anxiety, and social isolation. Social media provides a space to connect with others with the same condition, gain information and support, raise awareness, and challenge misconceptions. This study aimed to explore the social media experiences of adults with visible differences.

**Methods:**

An inductive qualitative design was employed, using online participant-driven, semi-structured photo-elicitation interviews with seventeen adults (14 female, 2 male, 1 non-binary) with a range of visible differences. Participants selected screenshots of social media posts which were used to guide the interviews. Reflexive thematic analysis was used to analyse the data and identify common themes, using NVivo 14 software.

**Findings:**

Three over-arching themes were generated: (1) Filtered realities: feeling self-conscious in a landscape of appearance ideals; (2) Developing my online self: a pathway to accepting my offline self; and (3) A place to belong: building visible difference communities online.

**Discussion:**

Adults with visible differences face similar appearance pressures on social media to the general population; however, the visible nature of their condition makes it more difficult for them to adhere to these norms. However, some had learned to use social media in a positive way to develop confidence and it provided a space to connect and gain advice from experts by experience. Participants felt that social media was a platform to increase representation of visible differences and normalise conditions; yet they acknowledged that balancing authenticity with content that received the most favourable engagement was a challenge.

## 1. Introduction

This qualitative study sought to understand the lived experiences of adults with visible differences in relation to their experiences of social media. Visible differences, also known as appearance-altering conditions or injuries, affect an individual’s appearance and make them look different to the ‘norm’ [1]. A significant number of conditions fall under the umbrella term ‘visible difference’, including those that are present at birth (e.g., cleft lip and/or palate, birthmarks, limb difference), acquired through illness (e.g., mastectomy), health conditions (e.g., eczema, lipoedema, alopecia), or injuries (e.g., burn scarring, amputation). Although visible differences can vary significantly in their physical symptoms, the psychosocial impact is similar across conditions and commonly includes anxiety, depression, low self-esteem, appearance concerns, intimacy concerns, life disengagement, and social isolation [2].

Social media consists of online platforms that allow people to build connections, interact in a shared environment, and convey their perspectives to others [3,4]. Social media is used for numerous purposes including communicating with others, for news, entertainment, educational purposes and, increasingly, sharing image-based content including photographs and videos [2,5,6]. Having risen to prominence in the 2000s, social media use is now extremely widespread, with an estimated 3.96 billion active users worldwide (7). For instance, in the UK, there are 56.2 million social media users, equating to 82.8% of population, with 73% of users engaging with social media every day [7]. Some of the most popular social media sites as of 2024 include Facebook, Instagram, Snapchat, X (formerly Twitter), YouTube, and TikTok. As social media forms such a central part of many people’s lives, it is important to recognise the role social media can play for adults, including those with long-term conditions including visible differences.

In addition to typical day-to-day use, individuals with visible differences may also use social media in relation to their conditions or injuries. This includes gaining information, connecting with others for advice and support, raising awareness of their visible difference, and fundraising for charitable and research-related organisations [8–10]. One central way adults with visible differences use social media is to access online support groups, often via the platform Facebook, which allow them to connect with others with the same condition. A qualitative study carried out by Iliffe & Thompson [11] found that online support groups for alopecia (i.e., hair loss) were beneficial for sharing experiences and advice, improving self-confidence, and increasing self-acceptance. Social media-based support groups for skin conditions have been found to provide a convenient platform for accessing condition-related information and support, which reduces access barriers, and can help form a sense of community, reduce isolation, and encourage helpful coping strategies [8].

Furthermore, online discussion boards such as Reddit (virtual communities where individuals can connect through posted messages) play a useful role in enabling individuals with visible differences to build community networks [12]. For example, individuals with craniosynostosis (a congenital condition affecting skull and brain) reported using them to connect with others and cultivate discourse about treatment options and experiences [13]. This research has also highlighted the importance of being able to access accurate information to prevent high anxiety in those with visible differences.

A small number of research studies have highlighted the social media experiences of individuals with visible differences. Young adults with burn injuries reported using social media to connect with friends and other people with burns, and as a mechanism for constructing identity [14]. Peer support websites have also been shown to provide social support and a place to rebuild after a burn injury [15]. Similarly, Stock and colleagues [10] found individuals with cleft lip largely focused on sharing experiences and seeking guidance from others via social media. Increasing a sense of community and connectedness and reducing isolation are common highlights from across the current research [16,17]. Taken together, these findings suggest many adults with visible differences utilise social media as a means of communication and connection with others, especially other individuals with the same or similar conditions. Carrying out more in-depth research using an inductive qualitative design would enable a better understanding of the overall experiences of adults with visible differences in relation to using social media.

In addition to the potential benefits, social media has also been shown to expose individuals to negative experiences. One study by Jones and colleagues [18] investigated the prevalence of cyberbullying for individuals with craniofacial conditions throughout adolescence by gathering retrospective perspectives from adults. They determined that nearly one third of the participants had experienced bullying on social media, particularly on Facebook. These findings indicate that negative experiences and instances of cyberbullying are common for individuals with visible differences, and that exploring lived experiences may help to understand and address these occurrences more effectively. Many of these findings concur with the wider literature investigating social media experiences for individuals with other long-term physical health conditions or disabilities. Additionally, within the general body image literature, there is a plethora of research finding that viewing idealised image-based content on social media, which often reflects narrow sociocultural appearance ideals, is associated with negative outcomes including body dissatisfaction, low self-esteem, and negative mood [19]. Moreover, highly image-based platforms such as Instagram and Snapchat have stronger associations with negative body image and using these platforms can lead to body dissatisfaction because users are encouraged to make social comparisons with peers and content creators [19].

Extant research helps to build a picture of what individuals with visible differences use social media for; however, to date there has been no detailed qualitative research exploring the lived experiences of adults with a range of visible differences in relation to using social media. Moreover, much of the existing research has focussed on support groups rather than looking at overall experiences of using social media as a person with a visible difference and has been limited to Facebook. While Facebook is still a widely used platform, others including YouTube, Instagram, and TikTok are now extremely popular, particularly with younger adults [20]. Most individuals use multiple platforms, which are found to have different uses to Facebook, such as sharing image-based content and carrying out activism, which are relevant to people with visible differences [20]. Therefore, it is important to explore general social media use within this study, rather than focussing on one particular platform.

In summary, most current visible difference research focuses on the type and accuracy of information available on social media, as well as literature examining social support, connecting with others, and raising awareness. However, there is little research specifically exploring how adults with visible differences experience social media. Additionally, current work predominantly focuses on Facebook, despite many people also using other, highly image-focused platforms. To address these gaps, the aim of this study was to qualitatively explore the experiences of adults with visible differences in relation to their overall social media use.

## 2. Method

### 2.1. Design

An inductive qualitative design using photo-elicitation interviews and reflexive thematic analysis was employed to explore the lived experiences of adults with various appearance-altering conditions in relation to their use of social media. This approach was chosen because it allows participants to construct detailed accounts of their experiences from the ground up, meaning that insights and theories are developed from the rich, ground-level data provided by participants, rather than starting with a predefined theory or framework [21]. This method is particularly suited to understanding lived experiences in underexplored areas, as it enables a more nuanced and authentic representation of participants’ perspectives. The inductive approach was guided by relativist and social constructionist theoretical assumptions, which support reflexive thematic analysis. Relativist assumptions, emphasising the subjective nature of reality, and social constructionist perspectives, focusing on how social contexts shape understanding, both align with the use of photo-elicitation as a method that enables participants to visually convey their experiences. Reflexive thematic analysis complements these assumptions by allowing researchers to critically engage with their own interpretations and the participants’ narratives, fostering a deeper understanding of the complex, socially constructed nature of lived experiences. Participant-driven, semi-structured photo-elicitation interviews were used as the data collection method to facilitate discussions and enable researchers to understand participants’ experiences through their social media posts. Together, these methodologies provide a suitable framework for capturing and analysing the nuanced realities of participants’ lives. Participant-driven photo-elicitation interviews involve participants selecting images (in this case social media posts) that relate to the research question, which are used to facilitate the interview process [22]. There are numerous benefits of photo-elicitation, particularly when exploring a visual topic, including helping the participant to articulate their experiences, allowing the researcher to access the participant’s world, allowing the participant to set the research agenda, and helping participants recall memories that are linked to their experiences [23].

### 2.2. Researcher personal statement

The first author is a White, female researcher and social media user in her early 30s with experience of living with the skin condition eczema. She has over nine years’ experience of carrying out qualitative research with individuals with visible differences, including photo-elicitation research. The second author is a White, male researcher and social media user in his mid-20s with no personal experience of living with an appearance altering condition or injury. He has two years of experience of collaborating on research with individuals with visible differences. The third author is a White, female researcher and social media user in her early 20s with no personal experience of living with an appearance-altering condition or injury. She has just under two years’ experience of working on appearance-focused projects, including with individuals who have visible differences. The last author is a White, female researcher and social media user in her mid-40s with no personal experience of living with an appearance altering condition. She has over 20 years of conducting appearance-related research. The first and last authors developed the research design, interviews and analyses were carried out by the first, second, and third authors.

### 2.3. Participants

Seventeen adults (14 female, 2 male, 1 non-binary), aged between 18 and 68 years, who self-reported having a visible difference and being a social media user participated in the study. This fulfilled the recommended sample size of 15–20 participants for qualitative interviews [24]. We also used the concept of information power to determine when to cease recruitment by reflecting on the richness and relevance of our data, and how well it related to our research aims, throughout the data collection process [24,25]. Information power suggests that the more relevant information the sample holds, the fewer participants are needed, emphasising the importance of the content of the data over the quantity. [26]. A total of 244 people initially signed up to the study by completing a registration form on Qualtrics. The researchers emailed individuals inviting them to take part, ensuring that the sample reflected a range of visible differences and demographic characteristics. Due to the large number of people signing up, the researchers undertook screening measures to identify fraudulent participants (see Mistry et al., 2024 [27] for guidance). In total, thirty-two participants were invited to interview, and seventeen composed the final interviewed sample. Individuals were excluded if they were identified as being fraudulent (i.e., did not have a visible difference).

Of the sample, one was Asian, two Black, one Hispanic, and thirteen were White. Four were married, eight were single, two living with a partner, one divorced and one separated. Eleven of the sample were heterosexual, one homosexual, two bisexual, and one did not define their sexuality. Seven of the participants reported having a disability. Of those who registered an interest in the study, the research team contacted individuals purposively to ensure individuals with a range of appearance-altering conditions were included in the study including alopecia, burn scarring, cleft lip and/or palate (CL/P), congenial melanocytic naevi (CMN), craniosynostosis, Dermatillomania (skin picking disorder), ectodermal dysplasia (ED), eczema, Lichen Planus Pigmentous, lipoedema, lymphoedema, Neurofibromatosis (NF1), psoriasis, Scleroderma, surgical scarring, and vitiligo. A range of demographic responses were collected to ensure that diverse perspectives were represented, which is crucial for capturing a full spectrum of experiences and opinions [28]. Additionally, a purposive recruitment strategy was used to ensure the study included individuals that reflected a range of socio-demographic factors. This approach helps to ensure the experiences of all individuals are included in research and to identify and address disparities among different groups, promoting equity and inclusivity in research [29].

### 2.4. Patient and public involvement

Patient and public involvement (PPI) was utilised in developing the research aims, study materials, and design. The overall aim of the study was established as the result of a priority setting exercises with a group of charities who support individuals with a range of visible differences. Additionally, four PPI representatives were recruited through the mailing lists of relevant charities to provide feedback on the relevance of the research aims, the clarity of the information given to participants, including the study advert, information sheets, consent forms, and instructions for selecting social media posts, and the interview guide. Feedback from the PPI representatives was incorporated before the study was launched. The first interview was also treated as a pilot and discussed with the research team.

### 2.5. Procedure

Volunteer sampling was utilised, and recruitment was conducted through the social media and mailing lists of the Centre for Appearance Research (CAR) and through a group of 30 charities known as the Appearance Collective, who support individuals with appearance-altering conditions and injuries. Individuals could register their interest via an online form and were subsequently contact by the research team. The research team purposively contacted individuals with a range of visible differences and demographic characteristics (i.e., age, gender, ethnicity) to capture diverse experiences. Ahead of the interview, participants provided written consent via an online Qualtrics consent form and were asked to identify four social media posts and email screenshots of them to the interviewer to be discussed during the interview. The instructions given to the participants were to “pick screenshots of your own posts that (a) you feel is typical of what you post on social media, (b) you feel is less typical of what you post on social media (not your usually style of post), (c) your favourite post on your social media account, (d) a photo or post from your account or another account that you think shows positive representation of appearance-altering conditions”. Recruitment was carried out between 26/06/2023 and 25/10/2023.

Individual, semi-structured participant-driven photo-elicitation interviews were carried out by the first and second authors via the researchers' university-approved Microsoft Teams accounts. The interviews ranged from 35 minutes to 74 minutes. Online interviews were chosen because they reduced geographical barriers to participation, which may have prevented individuals who did not live in the local area from taking part. This was particularly important because the research was carried out in collaboration with national charities that support individuals with visible differences. The researchers obtained additional verbal consent at the beginning of the interviews. The participants were asked open-ended questions about how they use social media, what it is like to use social media as a person with a visible difference, and the benefits and challenges of social media. The interview was driven by the four social media posts selected by the participants. Specifically, the interviewer asked the participants to describe each social media post in turn, to explain why they had chosen it for the interview, and how it related to their experience of using social media as a person with a visible difference. Prompts and follow-up questions were used to elicit in-depth conversations about the posts (e.g., ‘Can you tell me more about?’, ‘How did that make you feel?’). The researchers met regularly to discuss the interviews and reflect on how their assumptions may have influenced the process. For example, they reflected that they had made assumptions that the participants might have largely negative online experiences (e.g., trolling, negative comments), which was likely due to their understanding of existing literature on the topic. They tried to be aware of this when carrying out the interviews and to keep their questions open, being led by the participants posts to overcome this.

After completing the interview, participants were emailed a £30 shopping voucher to thank them for their time. The interviews were video-recorded and transcribed verbatim by the Microsoft Teams transcription function and transcripts were checked and amended as necessary by the project administrator.

### 2.6. Data analysis

The interview data were analysed using reflexive thematic analysis (RTA), a flexible, inductive approach to qualitative analysis used to explore patterns of meaning within lived experience [30]. First, the authors familiarised themselves with the dataset by reading and re-reading the interview transcripts and noting down any initial reflections. Next, the first and second authors carried out initial coding on half of the dataset each using a shared NVivo 14 file. This involved developing semantic, linguistic, and latent codes to summarise all relevant interview data. Semantic codes summarised the basic content of the data, taking what the participants said at face value, linguistic themes focused on the language used by participants, such as specific choices of words or metaphors that conveyed meaning, and latent codes related to possible underlying meanings and interpretations. These codes were all analysed together in the next stage of reflexive thematic analysis and helped organise and interpret the data, providing a richer and more nuanced understanding of the research topic.

Following this, initial themes were generated by searching for patterns of meaning and grouping similar codes. The themes were then reviewed and refined by the first and second authors, and themes and subthemes were developed through identifying central organising concepts and patterns related to shared lived experiences. The themes were discussed with the rest of the research team, finalised, and named and written up into a narrative. Throughout this process, the first and second authors met to reflect on the analysis process, including the influence of their own experiences, and to discuss the themes and narrative. As a research team, we had experience of using social media but not in relation to having a visible difference (e.g., we had not used it to gain support, raise awareness of visible differences etc.). Therefore, we worked with adults with visible differences who used social media to develop the study including the methodology and interview questions and gain feedback on our approach. When carrying out the analysis, the first and second authors reflected that they found themselves naturally grouping the codes into positive or negatives; however, this meant that the themes were very surface level and did not reflect the nuances in lived experiences. Therefore, they went back to the data and tried to develop more semantic and latent coding and be guided more by the meaning of the participants’ experiences, rather than imposing broad and over-simplistic categories so the analysis remained inductive. Participants’ social media posts were used to guide the interview and elicit deeper discussion and were not analysed; however, descriptions of the screenshots are integrated within the results section where applicable.

### 2.7. Ethical considerations

Ethical approval for this research was granted by the university College Research Ethics Committee (reference: HAS.23.02.070). Participant interview quotes are included in the manuscript to support the themes. Due to ethical restrictions relating to anonymity, the authors cannot share a de-identified or anonymised version of the raw interview data. The collection and analysis method complied with the terms and conditions for the source of the data, in line with approval from the university Research Ethics Committee.

Guidelines on carrying out visual research methods were adhered to [31,32]. The authors did not want to prevent participants from freely selecting social media posts from their own accounts, or the accounts of others due to concerns that the photographs would be published; therefore, the social media posts are not included in the manuscript. Additionally, no descriptions of the chosen posts are included. Social media posts were emailed to the researcher and stored in a secure OneDrive folder only accessible by the research team. The posts were deleted from emails once they were transferred to the secure drive. Posts were saved anonymously, and data will be destroyed after 5 years in line with university regulations. All audio files and transcribed interviews were securely saved to the OneDrive and will also be destroyed after 5 years. Participants were informed of the aims of the research prior to the study on the Participant Information Sheet and were informed of the potential to discuss topics that may be sensitive. The Participant Information Sheet also provided potential sources of support. Before the interviews, participants were reminded they did not have to answer any questions that they did not want to, they could pause or end their interview at any point and could contact the researcher for further information about sources support. The research team has much experience of carrying out qualitative research with individuals with visible differences.

## Results

Participants reported using the social media platforms Instagram (*n* = 15), Facebook (*n* = 12), X/Twitter (*n* = 5), TikTok (*n* = 6), Snapchat (*n* = 4*),* LinkedIn (*n* = 3), Pinterest (*n* = 1), YouTube (*n* = 1), and Reddit (*n* = 1*).* Through reflexive thematic analysis, three over-arching themes were generated:

- *Theme 1. Filtered realities: feeling self-conscious in a landscape of appearance ideals.*
- *Theme 2. Developing my online self: a pathway to accepting my offline self.*
- *Theme 3. A place to belong: building visible difference communities online.*

**Fig. 1:**
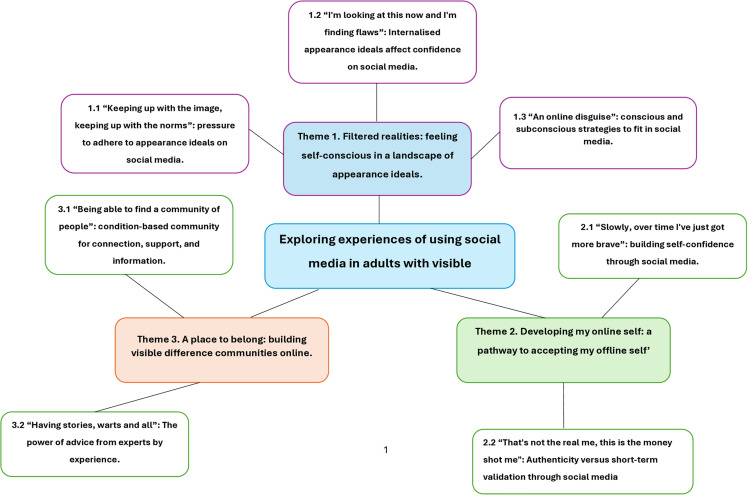
Thematic map – Exploring experiences of using social media in adults with visible differences.

The themes related to the anxiety and self-consciousness the participants felt about showing their visible differences on social media due to external and internal appearance pressures, the journey through which they had become more confident and saw social media as a platform for good, and the unique community that social media provides for people with lived experience to support one another. The themes and subthemes are outlined below with excerpts from the participant interviews and the use of pseudonyms. The themes are visually presented in Fig 1.

### Theme 1. Filtered realities: feeling self-conscious in a landscape of appearance ideals

The first theme relates to how the image-focussed landscape of social media leads to self-consciousness about posting content for people with visible differences, particularly in relation to showing their condition. This stems from the ubiquity of beauty ideals in the social media realm, including external appearance pressures from society, and internal pressures from having internalised these ideals, which led the participants to mask their differences both consciously and subconsciously. Theme one has three subthemes: *1.1 “Keeping up with the image, keeping up with the norms”: pressure to adhere to appearance ideals on social media, 1.2 “I’m looking at this now and I’m finding flaws”: internalised appearance ideals affect confidence on social media, and 1.3 “An online disguise”: conscious and subconscious strategies to fit in on social media.*

#### 1.1. “Keeping up with the image, keeping up with the norms”: pressure to adhere to appearance ideals on social media.

The participants felt significant pressure to adhere to beauty standards on social media; however, they felt that many elements of these standards (e.g., clear skin, symmetrical face) were incompatible with visible differences. This was particularly the case for image-based platforms such as Instagram:

*“Instagram…it creates this very high threshold in terms of appearance, in terms of perfection, in terms of what’s seen as attractive… if you have any visible difference, anything that makes you kind of ‘stand out’ will make it difficult to be that ideal”* (Phoebe, 38 years, dermatillomania).

Many of the unattainable beauty ideals that are pervasive in traditional media (i.e., television, film, magazines) have infiltrated social media, too. The participants felt that this created pressure for members of the public to attain these unrealistic standards and construct a ‘perfect’ online self:

*“Movie stars, anyone that’s wealthy is also a very good looking person…and you see it on social media. All influencers are all beautiful models, obviously. I mean, it’s that whole thing, right, where being beautiful is like the goal. It’s social media, [it] teaches you that you should be perfect. You should look perfect. You should have a perfect body. You should have a perfect face. You should have perfect hair. And it allows people to judge people based on those things - you judge people based on their looks because you’re not actually seeing them in person or talking to them in person. And that’s then that leads to the comments where you can say whatever you want because you’re not actually standing in front of that person”. (Kathy, 30 years, scarring).*

Here, Kathy suggests social media’s emphasis on perfection, along with the anonymity of interacting from behind a screen, actively encourages people to make appearance-based judgements and comments which would not necessarily occur in everyday life. Although the participants reflected that all social media users experience pressure to portray the ‘best’ version of themselves online, the culture “*sets an impossible task” (Lola, 36 years, scleroderma)* for people with visible differences because their appearance deviates from accepted societal norms. Therefore, constructing their online ‘self’ required significant time and effort and often involved attempts to hide or conceal aspects of their appearance that were not deemed socially acceptable:

*“I’m sure that all people feel that pressure, but people who live with a visible difference will have more of a challenge to strive for that because it means even more adjustments, even more hiding”. (Phoebe, 38 years, dermatillomania).*

Moreover, Catherine reflected that by re-shaping her online self so much, she had undermined her sense of identity in the real world. In the excerpt below, her use of the word ‘shell’ gives the impression that using social media can be a hollowing experience which erodes an individual’s identity:

*“I think that you can try and be something you’re not, and so therefore that sort of gets reflected in your everyday life. I think you can become so obsessed with your image on social media that in real life, it just a shell of your true self.” (Catherine, 22 years, eczema).*

It is even more difficult for adults with visible differences to fit online because social media is often used to perpetuate negative stereotypes, which increases stigma and discrimination towards people with visible differences:

*“Social media can maintain stigma in the jokes and throw away comments, often a meme to do with how ugly an individual is…I can think of numerous examples. One is a video of a man walking towards a veiled bride sitting on his bed, he unveils her to find someone made up to look ‘monstrous’. The reel is a joke about what your tinder date looks like when you meet them in real life, but [it] can play on the insecurities that people with visible differences have to live with when it comes to how to present to people online versus real life” (Daniella, 44 years, lichen planus pigmentosus).*

Here, the veil is a metaphor for the true self that is hidden on social media. Similarly, some participants explained that there are features within social media accounts such as Instagram, TikTok, and Snapchat that emulate facial differences for entertainment purposes:

*“There’s a lot of like body checking almost. So, using filters to see like is my face symmetrical? I’ve seen a lot which must be terrible for people who have other facial differences…like putting on a filter that’s like a funny, exaggerated proportion and then get off to see if you’re like pretty or not.” (Christine, 22 years, cleft lip and/or palate).*

The appearance pervasive landscape of social media including features that encourage users to enhance their images to reflect stereotypical appearance norms, placed external pressures on the participants and made them feel that, as individuals with visible differences, social media was not always a place that they could fit in and be an active part of.

#### 1.2. “I’m looking at this now and I’m finding flaws”: Internalised appearance ideals affect confidence on social media.

The second subtheme relates to how the external pressure to adhere to beauty standards on social media led many participants to internalise appearance ideals. Consequently, they felt self-conscious about posting on social media when they felt unable to meet these standards and to over-scrutinise their appearance in posts. In Ali’s quote below, it is clear she has internalised online beauty standards from the way she believes that showing her burn injury online may lead to her losing followers:

*“I don’t want them to be like ‘ohh, she’s showing herself’ and I think I might lose some followers…some people might find it disgusting…because of the burns on my body and face”. (Ali, 46 years, burn scarring).*

Some participants also reported being hyper critical and judgemental of photographs of themselves, scrutinising any perceived imperfections or features they felt misaligned with online beauty standards. For example, when discussing a photograph of themselves, Taylor explained how even if they initially feel happy with a photograph, their insecurities will lead them to overanalyse it and find aspects they are dissatisfied with:

*“I like the initial photo, I’ll try and not think about it too much. Whereas, if I tend to look at it too much - like I’m looking at this now and I’m finding flaws - if I tend to do that too much, then I just end up…I end up looking at it too long and then I find problems with it” (Taylor, 22 years, CMN).*

Similarly, due to internalised pressures to have perfect symmetry, Kathy had become very self-critical because of the impact her condition had on her face. This made her feel ‘vulnerable’ and wary of using social media:

*“It’s like less selfies as I’m ageing, my asymmetry is getting worse, which I was kind of told would happen. So, I think I’m like a little bit more vulnerable now and I’m a little bit more judgmental of myself now”. (Kathy, 30 years, scarring).*

These concerns made many of the participants feel unable to portray authentic representations of themselves on social media, particularly because of fears they would be judged by others and receive negative feedback on their posts. Although a number of participants set their accounts to private, there were still concerns about data on social media being shared publicly and losing control of their own images:

*“Instagram is like you’re sending it out into the world, and it could be seen by everyone and a bunch of strangers...I don’t have the bravery or courage that other people do. But I would love to be able to post more photos of myself that aren’t edited or photos of myself that that share my differences like openly and willingly.” (Kathy, female, scarring).*

Moreover, Christine felt that she would not receive positive reactions if she posted photographs of herself on social media. However, rather than receiving negative attention and comments, she worried that she would receive no reactions at all because content reflecting ideals gets engagement online:

*“I guess selfies, for example, are things that people post when they feel like they look good, and they look pretty and they’re looking for the interaction of like the people who follow them to comment and tell them they look great and like give them that feedback. I know that I’m not going to get that feedback…that’s never something that’s happened to me and so, then it’s just posting a picture of myself to silence. And that’s really weird and odd and makes me feel bad. So that’s why I don’t do that.” (Christine, female, cleft lip and/or palate).*

However, some acknowledged that much of the criticism of their posts came from within:

*“A lot of the times…we have this preconception in our mind that people are judging our social media post in a way that they’re probably not. They’re probably just seeing it and then just scrolling on, moving on” (Rahul, 33 years, NF1).*

*“I know people are less likely to sort of complain openly to my face, but I think I’m more self-critical of the photo of myself” (Taylor, 22 years, CMN).*

These quotes suggest that it is perhaps the internalisation of these ideals, and fear of negative appearance evaluation, that prevents the participants from posting on social media. In conjunction with this, some reflected that many of their concerns may not be down to their visible difference, and are in fact the same pressures everyone faces when using social media:

*“There’s a lot of insecurities with even people that don’t suffer or don’t have the conditions that like I have that say, for instance, even people and I know this for a fact like people say “ohh I don’t like to show my ears because they’re too big” or “I have pimples”, “I have this, I have that” and they’re very self-aware” (Lola, 36 years, female scleroderma).*

In summary, the appearance ideals that saturate social media had impacted how the participants viewed themselves and made them feel self-conscious about posting and hyper-critical of any posts of themselves.

#### 1.3. An online disguise: conscious and subconscious strategies to fit in on social media.

The third subtheme relates to the lengths the participants went to conceal their visible difference to fit in with the appearance standards on social media, both consciously and unconsciously. The participants used several practical and digital techniques to disguise their difference online. For example, some participants were able to cover their difference through their choice of clothing:

*“They [thighs] are always covered, they’re in trousers or occasionally I might wear something that goes up to the knees, but I never wear anything like that shows the upper part of my legs” (Ada, 50 years, lipoedema).*

Interestingly, this was not always a conscious process for the participants. For instance, when looking for photographs to discuss with the interviewer, Taylor was surprised realise how often their birthmarks were covered:

*“It’s amazing going through screenshots of trying to find photos for this interview and realising how many I had my sleeves on” (Taylor, 22 years, CMN).*

In addition to using clothes to cover their conditions, some constructed their poses in ways that concealed their difference. This had been the case for Rahul, who had a condition that affected one side of his face:

*“This particular picture is taken from the right side. Showing the right side of my face, not the left because the left side of the face is where the tumours are growing, whereas the right side there’s no tumours and a lot of my pictures on social media typically work from the right-hand side just because I never wanted to show the world the left side (Rahul, 33 years, NF1).*

Interestingly, while only ever showing one side of your face is not something that can easily be achieved in the real world, the online space provided an opportunity to portray himself in a way that masked this part of his identity. As well as using physical means to conceal their condition, the participants made use of digital tools to align more with specific beauty standards (e.g., clear skin, larger eyes), including digital photo-editing tools and so-called ‘beauty filters’ that are built into many social media sites. For instance, apps where you can *“remove all the acne, all the spots, or the scabs, all the scars, you can change the tone of the skin, you can add eyelashes*” (Phoebe, 38 years, dermatillomania). These practices were commonplace for several of the participants:

*“I hate how I look in front of a camera…I always post edited [photos] (Kathy, 30 years, scarring)”.*

*“It’s got a very strong filter on it, which is basically air brushed my face. Probably made my eyes look a lot bluer than they are and, yeah, it’s very heavily filtered” (Catherine, 22 years, eczema).*

The way Catherine describes these tools as being ‘strong’ and ‘heavily’ filtering her appearance conveys the extent to which individuals change their appearance to feel comfortable and acceptable on social media. Although participants reflected that the time and effort was often not worth the ‘reward’ of gaining positive attention through likes and comments, the external pressure made them feel compelled to continue:

*“In terms of Instagram [there is] a lot of pressure kind of making you feel bad on [the] inside, that I will never be this unless I use some filters then maybe and hide some of the features and a lot of effort going into ‘how do I pose for a picture to even start with?’. So, it’s a lot of pressure. It really is for this little reward, but it clearly is something if we are doing it for positive feedback from others.” (Phoebe, 38 years, dermatillomania).*

Participants also felt more comfortable in photographs with others or when the content they posted portrayed them in a humorous or non-serious way:

*“In photos, I usually pull faces to sort of like, not hide the difference, but to like make it ironic so that if I take a photo and I notice the difference, but I’m pulling a face, to me it makes it more like ironic and I can justify it… I’ve never actually psychoanalysed this before, but like when I pull that face it kind of makes the difference look more ironic as opposed to an actual problem with me.” (Lily, 23 years, craniosynostosis).*

Here, Lily reflects that by pulling a face she is accentuating her difference in a purposeful way, so that it becomes “*very apparent that I’m making the difference*”, which gives her control over how she is portrayed and perceived by viewers. This suggests to those viewing the photograph on social media that she is deliberately opposing beauty norms, and the intention is for the photograph to be non-serious and ‘ironic’. This may be a protective mechanism which deters others form judging her based on beauty standards.

In summary, this subtheme relates to the extent to which participants would attempt to disguise their difference to fit in with the culture on social media; however, this involved covering or hiding a part of their identity.

### Theme 2. Developing my online self: a pathway to accepting my offline self

The second theme relates to the way that many participants had overcome their self-consciousness around using social media and learnt to accept their condition and become more comfortable with it. This included becoming more confident being authentic on social media, which helped them to become more confident offline. This theme has two subthemes: *2.1 “Slowly, over time I’ve just got more brave”: building self-confidence through social media* and 2.2 *“That’s not the real me, this is the money shot me”: Authenticity versus short-term validation through social media*.

#### 2.1. “Slowly, over time I’ve just got more brave”: building self-confidence through social media.

The participants’ relationship with social media was complex: while it could be a place that made them feel self-conscious and insecure about their appearance, it could also provide a unique platform for them to gradually become more comfortable with exposing their visible difference. Specifically, it was a space for them to slowly increase their confidence and self-expression. When discussing how she started sharing photographs and personal stories about her eczema on social media, Catherine explains:

*“I think that it was a slow process because it was a process of your confidence growing and just a bit of self-love. But it was definitely the start of my posts changing and then being a bit more authentic…I think you’re completely trying to break a cycle that you’ve been in for a really long time” (Catherine, 22 years, eczema).*

For Catherine, social media provided an environment to sew the seed of confidence and watch it grow over time. This slow, nurturing process was also experienced by Amelia, who gradually built up to posting more about her psoriasis after being inspired by others who had allowed themselves to be vulnerable by showing their conditions online:

*“I think when I was like really shy at the start about posting like, ‘ohh, I think I didn’t want to post too much’ and I was a bit careful about like the sort of pictures I put up, because like say if there was like a picture where like my psoriasis looked quite severe and I didn’t want people to think that’s a bit gross if I post that. I think it was just seeing other people post like vulnerable things. I was like ‘ohh, they’ve done it, so I can do it’ and then I think over time I’ve just got more um, yeah, more confident with it.” (Amelia, 26 years, psoriasis).*

Importantly, the participants reflected that becoming more confident on social media had enabled them to be more accepting of their condition, and comfortable showing it, in their day-to-day lives. For example, Rahul discusses becoming more confident posting pictures on social media that show him when he is not wearing his prosthetic eye:

*“To start off with I was very shy on social media and I always used to put photos of me from my right-hand side. So, my ‘better side’ - you know to quote - and I’d always just do that, and I’d never put a front on picture because I’d feel that it’s too ugly to put a front on picture or that people will judge me or people will comment…but as time’s gone on and I’ve learnt to accept myself and this is a story I share with people on social media, now is that before you can expect others to accept you and like you and think…you’ve got to accept and love yourself and that’s the mindset and the transition I went through and now, yesterday I posted a video on TikTok without my eye.” (Rahul, 33 years, NF1).*

Rahul reflects that to feel accepted by others it is necessary to accept yourself. This links to the way that having the confidence to portray yourself in a positive and authentic way online can have an impact on how others see and view you. The interactive function of social media, which enables users to give feedback through likes, reactions, or comments, was perceived in both a positive and negative light. Although participants were fearful that they would receive negative attention, they also reflected that positive feedback could be reassuring and encourage them to continue expressing themselves authentically online, helping their confidence grow:

*“Loads of people comment like love hearts and stuff like that. And like loads of people just be like really nice and, yeah, they’ll be really nice, and they’ll comment really nice stuff, which yeah, is good…It helps me quite a lot and it helps me like it gets me to a point where it’s mentally like I’m mentally doing OK” (Jessica, 21 years, ectodermal dysplasia).*

In addition to this, Amelia explained that receiving support and positive reinforcement from gaining followers and the engagement she was getting on her posts helped to facilitate this process:

*“I think it’s just I’ve slowly overtime I’ve just got more brave with it and I think when I got like I noticed I was getting like quite a few like more followers and stuff and I was getting like a sort of like not opportunities but like sort of people were reaching out like companies were asking to send like products and stuff like that. And I was a bit like ohh, this is actually, I’m actually doing a good thing I could yeah, I could be a bit more brave about it” (Amelia, 26 years, psoriasis).*

Although it was not always easy to portray their authentic selves on social media, the participants felt that it was worthwhile and would have significant benefits for their wellbeing and to help others with visible differences:

*“For individual people like myself, who actually puts yourself out there because the more you hide, the more you would be a recluse. You’ve got to push it hard, you know, yeah everyone has crap days. But some have worse days than others. And yeah, you you’ve got to push and get your face out there (Tom, 42 years, burn injury)”.*

The way Tom describes ‘pushing’ illustrates how being present on social media can be a struggle but that it is worth the fight.

In summary, many of the participants had started to overcome the challenges of feeling self-conscious on social media and found that, in turn, this made them feel more confident about their visible difference when they were offline, in their daily lives.

#### 2.2. “That’s not the real me, this is the money shot me”: Authenticity versus short-term validation through social media.

Even when the participants did feel more comfortable posting their visible differences on social media, they were not immune to social media-related pressures. Specifically, many felt torn between wanting to portray themselves and their visible difference authentically and posting in a way that would lead to external validation. For example, Daniella describes how she is always cognisant of engagement from other people on her social media accounts:

*“I feel like it [social media] engages a more superficial part of me that’s worried about how people perceive me, and who likes my posts” (Daniella, 44 years, Lichen planus pigmentosus).*

Furthermore, the portrait of perfection is not limited to appearance on social media, and the participants felt pressure to portray many of aspects of their lives in a positive, often unrealistic, way:

*“What most people do, they try to look - you try to look really happy. You try to look like you don’t have any major troubles. You look at you, you try to create almost like a double life on the internet. You just try to look like everything’s better than it really is” (Kathy, 30 years, scarring).*

*“I think that we’re as a society anyway, we really struggle with likes and you know, people thinking we look good and portraying ourselves on social media to have this perfect lifestyle, even if we say we don’t, I think we do sometimes, so growing up with a visible difference, you’ve got that on top of already the normal stress of going ‘I’ve got to put a filter on this cause my face is bright red’ or ‘what if this person notices this?’ It’s really hard because you want to be raw and you want to be transparent and that’s what social media is now, but it isn’t, and the filters are just getting more real looking so it’s really difficult. And I think there’s added pressure of growing up on social media when you’ve got a visible difference” (Catherine, 22 years, eczema).*

Catherine describes how social media should be real or ‘raw’; however, it is often a distorted depiction of reality, whereby so much of it is built on tools to make your life look better that it is very difficult to be transparent and authentic. In addition to this, social media functions by enabling engagement and connection between users, including through liking, reacting, sharing, and commenting on other people’s posts. Although the participants had discussed how this feature encouraged them to be more confident sharing photographs of themselves (see theme 2.1), some acknowledged that their posting behaviour was ultimately driven by the desire for external validation:

*“I think it’s always, you know, as much as we pretend that external validation is not necessary, I think that’s always good to, it makes you feel good to get external validation that your photo’s nice…I was always posting a picture and if I in 24 hours sometimes I hadn’t got enough likes. Sometimes I take the picture down, I just delete it” (Rahul, 33 years, NF1).*

Phoebe discusses this phenomenon being a ‘double-edged sword’: although she gains initial positive reinforcement when posting an image that conforms to these standards, she later feels guilt over the in-authenticity:

*“I think it feels good in the moment. It feels good to get this nice feedback afterwards, but then actually makes a feel really bad because you’re thinking that’s not the real me, this is the money shot me… It’s not who you’re going to see in in the morning when they wake up… So it’s a kind of double edged sword” (Phoebe, 38 years, dermatillomania).*

Although these pressures were persistent, some of the participants had overcome them and felt that being authentic was more important than receiving external validation:

*“I used to heavily filter my photos but like now I realised that it’s like portraying, it’s like making young women more mentally ill because of like how much celebrities filter the photos and I’m not going to live my life, my life as a lie. And I put, like, put myself in social media as, like an honest person”. (Jessica, 21 years, Ectodermal dysplasia)*

In summary, the participants often felt caught between wanting to gain external validation and being authentic online; however, a number felt that the benefits of being positive role models and portraying themselves realistically outweighed the need for validation and helped to present people with visible differences in a positive way more broadly.

### Theme 3. A place to belong: building visible difference communities online

The third theme relates to the unique space social media provides for individuals with visible differences to connect, share experiences, and gain advice and support. This has three subthemes: *3.1 “Being able to find a community of people”: condition-based community for connection, support, and information, 3.2 “Having stories, warts and all”: The power of advice from experts by experience, and 3.3 “You can be an ambassador for what you look like, but you don’t have to make that your whole personality”: feeling a duty to advocate online.* The participants reported joining groups and following visible difference influencers to find information about treatments, provide and receive support, and to partake in advocacy and awareness raising themselves.

#### 3.1. “Being able to find a community of people”: condition-based community for connection, support, and information.

Most participants were members of, or engaged with content from, condition/injury-related communities on social media. Participants described social support, information on their condition and treatment options, and sharing or reading personal stories as being the main functions of these communities. Many of the participants had originally felt isolated and different from their peers because of their condition and, especially for those with rarer conditions, did not know anyone else who shared their experiences or *looked* like them. Amelia explains how she felt very lonely when she thought she was the only person with psoriasis but, by sharing her experiences online, she came to see many other people have the condition and she found people she could relate to:

*“I think any condition or any visible difference can be really lonely and if it’s something that is also quite rare as well…. none of my friends had psoriasis and I was really embarrassed because I had it on my scalp and then it started to come all over my face, which is obviously quite erm hard to hide that...and I know it sounds really silly now because I know there’s so many other people that do have it, but at one point I genuinely thought I was like, the only person in the world who had, like, psoriasis on my face, cause I just never knew anyone else who did. And so that’s why I started my Instagram in lockdown… and that was when I saw so many other people who were like me and I was like, ‘ohh wow, there’s like so many other people who have psoriasis’ and it’s like a really common thing and I just didn’t know. (Amelia, 26 years, psoriasis).*

For Amelia, social media opened a new world of people which allowed her to *“find[ing] people to talk to and when other people don’t understand. Obviously…my friends don’t have it, my family don’t have it. It’s just nice to talk to other people”.* Having been misdiagnosed for many years, Sandra also felt like she was the only one who looked like she did and always had her experiences dismissed by others. However, joining an online support group enabled her to meet other women with lymphoedema and eventually see other people who looked like her:

*“It started as an online community, but people have got together... [they are] just a group of women like myself who and one of the ladies there she set up a meeting two or three times a year…I walked into this room with the Lipoedema group and they were all these people who looked exactly like me” (Sandra, 68 years, lymphoedema).*

It can be seen here that being able to foster real-life connections from meeting people online was an important time for Sandra because it allowed her to finally see other people who looked like her. In addition to not feeling alone, online communities also provided emotional support:

*“It’s, it’s supportive. It’s just like people like saying nice things like. I don’t know, just being there, just being like ohh I’m here if you want to talk or I understand what that’s like. And ‘ohh I tried that it didn’t work for me either’. Or that sort of thing. It’s just nice. Yeah, it’s just nice to like have, like I say, like that sort of community to talk to” (Amelia, 26 years, psoriasis).*

On the other hand, rather than gaining specific support or interacting with others, Jasmine used social media to explore presentations of her condition, vitiligo, in a positive way.

*“I personally like just like picture ones instead of like reading experiences or whatever. And it sometimes just like a really artistic like photography picture of someone with like vitiligo and it’s like in black and white or they’ve got like paint splashed on them or something or something where it literally looks like artwork of where they’ve almost enhanced pictures for the better or yeah, I was really into like researching at one point of like fashion shows and like other stuff where people, it’s almost one of those visible differences that is like really interesting to look at and it’s not, it’s nice for me to look at it on other people as well and just whether it’s like sense of community or something, but to just see it on other people and and see it like in my opinion, look like really cool as well and like different like patterns and everything. (Jasmine, 18 years, vitiligo)”.*

Overall, the online communities helped people connect with others who had shared experiences and looked like them. This made them feel like they were not alone and allowed them to gain emotional and practical support. This was particularly beneficial for individuals with rare conditions who may not be able to meet one another in person.

#### 3.2 “Having stories, warts and all”: The power of advice from experts by experience.

A central aspect of visible difference communities on social media was their function as a platform to share and receive condition and treatment related advice and information from/with others with similar experiences. As Christine explains, the nature of social media, whereby content is generated by members of the public, means much of the content features posts from people sharing personal experiences of health conditions rather than the factual, scientific focus often shared in traditional media, which focuses on the condition, rather than the person experiencing it:

*“Social media is very much built on sharing your experiences…it’s just a very convenient medium to share your personal experience, instead of just the, I don’t know, the very harsh science of it” (Christine, 22 years, cleft lip and/or palate).*

Another benefit of social media groups relating to different visible differences was that they showed the reality of conditions which helped prepare an individual when they were at the beginning of a journey and wanted to find others they could relate to:

*“There’s often groups of happy looking people without their wigs on all smiling and wearing the same T-shirt, you know, at an event or whatever, which is great. But when you’re in the mix or midst of it all and you’re trying to navigate stuff, you can’t ever imagine being that person…You know, and having stories, warts and all, you know, is I think quite powerful.” (Zoe, 49 years, alopecia).*

The way Zoe describes the ‘warts and all’ suggests that these groups are helpful by laying the condition bare and helping her to understand what the experience can really be like. Similarly, Daniella found that she was able to connect with others with the same rare condition through social media and gain personal feedback and advice relating to potential treatment options:

*“I found I was able to connect with people who had this condition by using hashtags, and given it is quite rare in the UK, it was good to be able to find people who are dealing with the same issues, including people on the same medical treatments with whom I could discuss side effects and how they manage them” (Daniella, 44 years, lichen planus pigmentosus).*

In conjunction with this, Christine had created an account to specifically document her surgery for cleft lip and/or palate to help prepare others. Sharing her story had a two-way benefit whereby she was able to help others, and she gained a sense of pride from being able to help others through her own challenges:

*“It felt really good to have an impact on other people who can be better prepared for what it was going to be like for them.”* (Christine, 22 years, cleft lip and/or palate).

In addition to practical and treatment-related information, Amelia felt that understanding the impact of a condition on other aspects of people’s lives and their overall wellbeing was beneficial and something that was missing from treatment more generally:

*“I think its just talking to people about, you know how they’re feeling as well, like in other ways, like how it can affect other things, like your mental health or like your work or relationships. Yeah, that’s just so much that they just talk about on it. Treatments I guess, I think treatments and like progress and people’s journeys like the most I think people talk about” (Amelia, 26 years, psoriasis).*

As someone who shared their surgery journey on social media, Zoe felt conflicted about the experience. On one hand, she recognised the power of being able to provide practical guidance and reassurance to others in the similar situation; however, as social media is a public platform, she was also followed by others from their personal life:

*“It was very helpful because I would have a lot of people who had either been there or were getting ready to be there commenting on how either they’d experienced similar things and this is what helped, this is what I did, that type of thing. But it was also very uncomfortable, because people who knew me in real life followed the account. And like, yeah, I don’t know, there’s an inherent vulnerability of talking about I don’t know how this is like an appearance based thing, It’s a plastic surgery. And it’s weird to be like here are my insecurities that I’ve written down, and you can now read and keep in your mind” (Zoe, 49 years, alopecia).*

Although their experiences were, overall, positive, several participants also expressed some concerns about using social media for sharing condition related information. For example, Sandra spoke about how people, who are often desperately seeking support, might use social media groups to try and gain a diagnosis of a condition from others with it:

*“There’s also the downside of it is a lot of people posting photographs of their legs and saying, ‘do you think I’ve got lipoedema?’”. (Sandra, 68 years, lymphoedema)*

Indeed, social media is largely unregulated and therefore information, including about treatments, can be disingenuous or harmful. Overall, the participants gained benefits from being able to hear from others with lived experience, who could provide guidance beyond medical information. Sharing experiences could be rewarding and help others but could come at a cost of sharing with others who the information was not intended for and there were concerns about the regulation of information online.

#### 3.3. “You can be an ambassador for what you look like, but you don’t have to make that your whole personality”: feeling a duty to advocate online.

Many participants also used social media as a platform to engage with and post content that raised awareness of appearance altering conditions. However, despite wanting to be a part of positive change for those with visible differences, some experienced a conflict between feeling duty-bound to advocate for their condition, but equally not wanting their condition to define their online selves. Participants felt that social media provided a space for realistic and positive information about visible differences, which challenged some of the more stigmatising content on other parts of the internet:

*“It’s just about changing the information that’s already out there. I mean, I know that probably about 10 years ago when you Google ‘CMN’ (congenital melanocytic naevi) on Google Images, you get like horrific images of like children in like Non-Western countries that’s dying basically. And it’s just about sort of like changing the narrative of like, all right, well, yeah, it’s not going to look pretty if you’re having it removed but like, actually there’s like lots of other people now on social media who are all promoting it. (Taylor, 22 years, CMN).*

Moreover, Rahul expressed how mainstream media dehumanises people with visible difference by using them as character tropes for villains or victims. However, social media provides a user-generated space to challenge this and show that people with visible differences are not defined by their conditions:

*“There needs to be more content, more sort of information about this the visible difference community. More people telling their story, more people talking about their challenges, and more people talking about how we overcome that. The more we start talking about it, the more we start posting about it, the more people will see it and the more people who are actually we need to start normalising this. And I think media in general need to do a better job that needs to be more positive representation in films, in documentaries, in TV in general”. (Rahul, 33 years, NF1).*

Moreover, for many, social media provided a platform to “*promote a message of awareness and acceptance” (Phoebe, 38 years, dermatillomania)* of visible differences:

*“I think it’s really positive to show skin with acne that it’s normal to have, you know spots and scars… you often can feel that other people find you disgusting basically, whenever I had some active scabs or spots on my face, I was always very self-aware of how disgusted other people can potentially be with it because it involves blood and it involves puss and involves things like that just makes us naturally very often go ‘eww’. And it’s just there on your face so. And having when it comes to this photo, what it does really good is just that it shows that you can have those changes on your skin and that’s OK and it should be seen as normal (Phoebe, 38 years, dermatillomania).*

In relation to normalising the condition, Lola describes how she wants to show people that someone with a visible difference can still enjoy beauty practices such as wearing makeup:

*“I am beautiful and I just wanted to show like I’m just like another person who likes to wear makeup and likes to look nice for things…I don’t want to give the opportunity for people to patronise me, but at the same I try to see it to the positive thing that I could be, you know, like a good representation of me” (Lola, 36 years, scleroderma).*

The power of these posts can be seen in the quote from Daniella where she reflected that as well as challenging the perceptions of society, posts normalising visible differences also challenge her own internal biases towards difference. As a member of society, she is also vulnerable to internalising stereotypes:

*“It can be quite empowering to see people with visible differences being ‘visible’ and normalising their differences. I find these posts have a positive impact, not just on how I feel about myself, but on my attitude to other people with visible differences, which is less judgmental (Daniella, 44 years, lichen planus pigmentosus).”*

Some participants felt it was necessary to advocate and raise awareness in order to take ownership over ensuring that things change, and the next generation live in a more accepting world:

*“A next generation is going to be brought into this world and they need to be aware that there’s a lot of people who are different and then they deserve to have the same, you know. Same environment, same seat at the table, whatever you want to call it, but it’s just we’re all in here at the end of the day” (Lola, 36 years, scleroderma).*

On the other hand, some participants were reticent about advocating on social media because they felt it could become an all-consuming role and that even if they used their platform in a positive way, they could become embroiled in political and potentially negative online culture:

*“I don’t know if this is because my generation or whatever, but I get tired. I get tired of negative comments, regardless if it’s on my defence, as in the way I look…I get [to] a point that I get exhausted with the all the political things” (Lola, 36 years, scleroderma).*

Nonetheless, others expressed that it is possible to find a balance and *“you can be an ambassador for what you look like, but you don’t have to make that your whole personality” (Taylor, 22 years, CMN).*

Overall, the participants felt that using social media to raise awareness was a positive thing; however, some also expressed concerns that social media is often an echo chamber, and those who would benefit from understanding more about visible differences were not likely to view these posts:

*“The problem, especially with facial differences, is getting it to people without facial differences like spreading it enough for them to actually see the post?” (Zoe, 49 years, alopecia).*

In summary, this theme related to the power of social media in challenging stereotypes and stigma towards visible difference. Many of the participants felt that they should also use the platform in this way; however, using it to raise awareness as well as for personal reasons could be exhausting.

## 3. Discussion

This study used novel, participant-driven photo-elicitation interviews to explore the social media experiences of 17 adults with visible differences. It adds to existing literature by providing an extensive and nuanced exploration of the experiences of adults with a range of visible differences and including a variety of social media platforms.

Although most participants (*n* = 12) did use Facebook, Instagram was most used (*n* = 15) and a third of participants also used TikTok and X (formerly Twitter). Echoing findings from previous research, the participants reported using social media to connect with others with their condition to gain treatment-related information, advice, peer support, and to view and share personal experiences and stories [8–10]. Moreover, social media was particularly beneficial for individuals with rare conditions because it afforded them the opportunity to meet others who understood their experiences and the challenges they faced, which helped them to feel less isolated [9,33]. One key benefit was that social media could be used to gain valuable information and advice from experts by experience, which went beyond educational and treatment-related information typically provided by the health service and support organisations. For example, some of the posts showed the reality of living with and undergoing treatment, provided more nuanced and relatable information, and gave practical information and tips that are not available in standard educational information.

This study highlights a key benefit of social media is being able to gain condition and treatment-related information; however, it is vital to consider the potential harms of medical and treatment-related advice shared by non-experts [8]. Indeed, treatment-related information is often produced by individuals with no medical qualifications, and inaccurate information is commonplace [34,35]. One example comes from Finnegan and colleagues [36], who identified misinformation about topical steroids (treatment for skin conditions) is prevalent on Facebook, Instagram, TikTok, and X, and alternative treatments with limited evidence of efficacy, including those that can be harmful, are often promoted. This is particularly concerning because this misinformation can negatively impact condition management, including leading individuals to cease medical treatments without support [16]. There are systemic issues with social media which perpetuate this issue. For example, social media content is largely unregulated and therefore many instances of misinformation are not identified and corrected. This issue is increasing given that in 2025 the social media company Meta moved away from employing fact checkers to relying on artificial intelligence (AI) [37]. This is particularly problematic because misinformation is highly prevalent across social media platforms and this type of content is found to receive greater engagement from users, meaning it has a widespread negative impact on users and is shared with vast numbers of people due to the algorithms used by the platforms [38].

Given that many individuals use social media for this kind of information, it is paramount that support organisations and health professionals work alongside social media companies to understand how to best support individuals with visible differences and protect them from harm. This may include health professionals creating social media content about treatments in collaboration with content creators who share their lived experiences [33,39]. One example of this is the YouTube ‘Health Shelf’ initiative, whereby experts create evidence-based content relating to different health conditions [39]. Furthermore, Bautista and colleagues recommend the use of ‘digital first responders’ who are trained to identify and correct misinformation online [40].

A key challenge for the participants was that much social media content echoes the narrow and unrealistic sociocultural appearance ideals that dominate traditional media (e.g., films, television, magazines). This sets the precedent that social media users must construct a ‘perfect’ online self, characterised by features such as flawless skin, facial symmetry, large eyes and lips, and a slim physique, particularly on image-based platforms like Instagram. This aligns with self-presentation theory, which explains how individuals seek to control others’ impressions of them through their self-presentation [41]. On social media, individuals may perceive a user to present a true reflection of their self-identity; however, it is often a curated version that emphasises positive aspects of their lives, including through the use of filters and editing tools [42].

These experiences also align with a plethora of research in the body image field, which show exposure to appearance ideals on social media is associated with numerous negative outcomes including body dissatisfaction, low self-esteem, and negative mood [43]. For example, the Tripartite Influence Model of Body Image [44] explains how appearance-focused media, such as social media, leads individuals to make appearance-based social comparisons with peers and content creators. This idealised content encourages people to evaluate their self-worth based on their appearance, often resulting in body dissatisfaction when they do not feel that they meet sociocultural beauty standards [45].

While these pressures are not unique to adults with visible differences, the participants expressed that these challenges were even greater for them because having an appearance that deviates from societal ‘norms’ makes it even harder to meet these standards. To achieve this, the participants employed various strategies, including posing from angles that disguised their visible difference, and utilising filters and beauty editing tools (which are built into many platforms) to emulate clear skin and symmetry but were often dissatisfied with the outcome. Again, in line with this, research finds that taking, and particularly editing, selfies leads to appearance dissatisfaction [19]. Therefore, these findings extend current understanding by illustrating that individuals with visible differences feel the same appearance pressures from social media; however, they experience additional challenges due to having a condition that impacts their appearance and may be difficult to conceal online.

Interestingly, although social media could make the participants feel self-conscious and draw attention to their appearance, it could also help them to become more confident and accepting of their visible difference. Specifically, social media provided an environment where they could gradually become comfortable with presenting their appearance difference. Seeing and interacting with others with visible differences on social media had often encouraged them to do the same and reassurance from likes and comments on their posts had led some to become more confident showing their visible difference offline, too. This aligns with findings from Iliffe and Thompson [11] who found that online alopecia support groups helped individuals to feel more confident and accepting of their condition. However, in the current study, presenting themselves authentically on their social media profiles allowed the participants to receive reassurance from connections without visible differences as well.

The participants also voiced that it was important to portray themselves authentically to dispel misconceptions and stigma towards visible differences and to challenge current appearance-related rhetoric on social media. Interestingly, although some reflected that their motivations for posting online were to receive external validation, which was more commonplace when posts reflected appearance ideals [19], the importance of presenting themselves in an authentic light outweighed this and some felt a duty to represent visible differences within society. Similarly, adolescents with the birthmark condition CMN in Guest, Williamson and Harcourt’s [23] study felt that having adjusted to their condition, they had a duty to help and support others. Nonetheless, some struggled with balancing advocacy and the rest of their identity and found it difficult to pursue both online. This may reflect the way that in *real* life individuals can present multiple identities, which may differ across situations, whereas online this is less nuanced, meaning it is difficult to encompass multiple aspects of identity. This may mean that having a visible difference is always at the forefront of their online identity and impacts how they can present themselves, and are perceived by others, online. Additionally, using social media for raising awareness and advocating can leave individuals vulnerable to negative comments and engagement, particularly for those who have public profiles and larger followings, and can put pressure on them to provide constant support to users who reach out to them for advice. These experiences can have a significant negative impact on an individual’s mental health [46]. It is important to understand these pressures in more detail and for social media users with visible differences to feel able to put their own mental health first. It would be beneficial to carry out further research with individuals who use their accounts in this way to understand their experiences and how best to support them.

One unique feature of social media (compared to ‘traditional media’) is the ability for users to generate their own content and increase the representation of appearances that are typically marginalised, such as visible differences, and challenge stereotypes [47]. There are an increasing number of accounts on social media that also aim to educate, raise awareness of, and reduce stigma towards visible differences and some have amassed large followings from the general population [47]. For example, Nikki Lilly, a young woman with the condition atrioventricular malformations (AVM) uses her social media platforms to raise awareness of appearance difference and spread messages of acceptance of appearance diversity and has 570,000 followers on Instagram and 2.9 million subscribers on YouTube. Positively, experimental research suggests that viewing social media content that represents people with visible differences, provides education, and challenges appearance ideals, can decrease stigma towards them and improve how a viewer feels about their own body [39,48]. However, a key issue with this is that algorithms on platforms including Instagram, and particularly TikTok, favour content that portrays appearance ideals [49]. Therefore, content raising awareness of visible differences is less likely to achieve significant reach and is more likely to be viewed by those who are actively seeking out the content, and are already accepting of visible differences, rather than those who may benefit most from it [39]. Given that platforms like TikTok and Snapchat are particularly popular in younger adults and adolescents, it is important to explore their experience including how they might differ by platform.

An additional and increasing issue for adults with visible differences using social media is the use of AI. For example, the inaccuracies of AI detecting and removing harmful content (e.g., hate speech and trolling), and reports that that AI mistakenly flags photographs of individuals with visible differences as harmful content, providing ‘content warnings’ or removing the posts [50]. This increasing reliance on AI, along with the prevalence of hate speech on the platform X (previously Twitter), which is often not effectively dealt with [51], leads to exclusion of people with visible differences from online spaces and perpetuates discrimination and marginalisation.

### Methodological considerations

This study has provided a rich insight into an under-explored area. As with other research into visible difference, the use of photo-elicitation was particularly useful when exploring topics relating to appearance and it helped to situate the participants’ experiences of using social media [23]. Moreover, the screenshots provided a frame of reference and allowed the participants and researchers to collaborate closely and for the participants to set the agenda for the interview [52]. On the other hand, it is also important to consider that some individuals may not feel comfortable in sharing their photographs for research purposes, which may have deterred them from taking part. Another consideration is the over-representation of female participants in the sample. The research team did employ strategies to engage male participants; however, it may be that the design of the study and use of photo-elicitation deterred some participants from taking part. Moreover, men are known to use social media platforms differently to females, such as preferring anonymous discussion-based forums such as Reddit over photo-based platforms. Further to this, although their experiences of using social media varied, and the researchers made an effort to recruit participants with a range of experiences, the findings may not be transferable to all individuals with appearance-altering conditions, including those who consume but do not actively post on social media. It is also possible that individuals who had more positive experiences of social media would have been more likely to participate.

While the social media experiences of the participants in this study were generally positive, it is important to note that individuals with visible differences can experience negativity including harmful comments, trolling, and even hate speech online. Many of the participants in this study had private accounts which enabled them to control their interactions with others, whereas other users and content creators with visible differences who have public profiles may be subjected to more negative experiences. It is important to consider both the benefits and potential harms of social media in this area.

### Research recommendations

This study has provided an insight into the experiences of adults; however, similar research should be carried out with children and young people with visible differences. This is particularly important because 91% of 12–15-year-olds in the UK use social media and young people are found to engage with social media differently to adults, including using more video-based platforms [53]. Furthermore, researchers should explore more anonymous social media platforms such as the discussion-based platform Reddit, which are commonly used by individuals with visible differences. Utilising an online survey may be a more suitable method to achieve this. Finally, future research should explore the experiences of influencers or content creators with visible differences, which may help to understand how to ensure information on social media for people with visible differences is accurate and not harmful.

### Practical implications

The findings also highlight numerous practical implications. First, given that individuals with visible differences have a strong preference for using social media in relation to their condition, and hearing personal stories, it is important that health professionals engage with social media to ensure accurate information is available and to identify and correct misinformation [40]. For example, this could include joining a content creator for an Instagram Live Q&A or writing content to be shared. Additionally, co-producing evidence-based guidance for charitable support organisations and content creators on how to best support and engage individuals with visible differences would be a useful tool. Finally, actively engaging with content that presents a diverse range of appearances and unfollowing, blocking, or muting accounts that post content reflecting appearance ideals, known as 'digital pruning', is a helpful strategy that is easy to implement [54].

## Conclusion

In summary, the participants in the study faced similar challenges to all social media users in relation to feeling pressures to conform to narrow appearance ideals on social media and portray a ‘perfect’ online self; however, having a condition that affected their appearance made it more difficult to fit in online and led them to want to disguise their condition. Nevertheless, social media could also provide a space for them to increase their confidence in showing their visible difference which translated into confidence in the offline world. Some of the key benefits of social media were being able to gain information and support from experts by experience, who had a unique understanding of the condition and could provide guidance beyond that of medical professionals. Finally, the participants felt social media was a place where visible differences could be normalised and the platform’s use as a place to advocate was seen as important; however, some felt torn between wanting to advocate and not wanting their condition to define their online presence.
